# Real-world national trends and influencing factors preference of non-vitamin K antagonist oral anticoagulants in China

**DOI:** 10.3389/fmed.2023.1258536

**Published:** 2023-11-21

**Authors:** Shaojing Zhu, Mengjie Li, Lujun Wang, Lu Hou, Dandan Li, Jiuhong Liu, Yanxia Lu

**Affiliations:** ^1^Department of Pharmacy, Medical Supplies Centre of Chinese PLA General Hospital, Beijing, China; ^2^Department of Information, Medical Supplies Centre of Chinese PLA General Hospital, Beijing, China; ^3^Department of Pharmacy, Beijing Friendship Hospital, Capital Medical University, Beijing, China

**Keywords:** NOACs, OACs, prescription pattern, AF, influencing factors

## Abstract

**Backgrounds:**

Non-vitamin K antagonist oral anticoagulants (NOACs) have been recommended as the first choice over warfarin for non-valvular atrial fibrillation (AF). However, there is limited data about their usage in mainland China.

**Methods:**

Prescriptions of patients diagnosed with AF and containing OACs were extracted from Hospital Prescription Cooperation Project from January 2016 to March 2021. The primary outcome was the changing percentage of different OACs. The secondary outcomes were frequencies as well as factors with the choice of different OACs and dosage of NOACs. Univariate and Multivariate logistic regressions were conducted to explore possible factors. All statistical analyses were performed using SAS software (Version 9.4).

**Results:**

Among the 220,083 distinct prescriptions diagnosed with AF and prescribed with OACs, the percentage of NOACs increased over years, exceeding warfarin in 2018. Until March 2021, 83.53% of included patients were prescribed with NOACs. Rivaroxaban (62.25%) and dabigatran (37.65%) were the most commonly prescribed NOACs. Low dosage was common for NOACs (44.54%), this was mainly driven by rivaroxaban, 67.98% of which were low dosage. Multivariate logistic regression indicated that several factors were positively associated with the preference of low dosage, including outpatients (OR 1.32, 95% CI 1.26–1.39), patients with hypertension (OR 1.49, 95% CI 1.40–1.58), acute coronary syndrome (OR 1.17, 95% CI 1.12–1.22), stroke (OR 1.42, 95% CI 1.33–1.52), and kidney disease (OR 1.63, 95% CI 1.34–1.97), as well as concomitantly using antiplatelet agents (OR 1.52, 95% CI 1.40–1.66), and steroids (OR 1.76, 95% CI 1.50–2.07). On the contrary, they were less common in health insurance holder (OR 0.79, 95% CI 0.75–0.84), patients taking apixaban (vs. rivaroxaban, OR 0.39, 95% CI 0.18–0.81), dabigatran (vs. rivaroxaban, OR 0.01, 95% CI 0.01–0.01), edoxaban (vs. rivaroxaban, OR 0.36, 95% CI 0.23–0.55), diagnosed with heart failure (OR 0.87, 95% CI 0.81–0.93), deep vein thrombosis (OR 0.36, 95% CI 0.29–0.46), pulmonary embolism (OR 0.35, 95% CI 0.28–0.43), and peripheral artery disease (OR 0.68, 95% CI 0.55–0.85).

**Conclusion:**

The usage of OACs for AF was overall complying with updated guidelines. Low dosage was common for NOACs, further studies were warranted to verify its effectiveness and explore the underlying mechanism.

## Introduction

Atrial fibrillation (AF) is a leading risk factor of arterial thromboembolic events including stroke and systemic embolism (1, 2), and the oral anticoagulants (OACs) have a fundamental role in the management of AF (3). Non-vitamin K antagonist oral anticoagulants (NOACs) have shown non-inferiority to warfarin in the prevention of stroke/systemic embolism (4, 5) and a more favorable risk-benefit profile (6) for non-valvular atrial fibrillation (NVAF). Furthermore, patients with NOACs demonstrate higher persistence compared with warfarin (7). However, though the Chinese guideline has also recommended NOACs as the first choice over warfarin in NVAF in 2018 (8, 9), it is not known whether this has been applied in general practice.

At the same time, low-dose of NOACs is reported to be frequently used in daily clinical practice (10). Though meta-analysis based on phase III clinical trials suggested that NOACs were more effective and safer in Asian than non-Asian (11), this phenomenon seems to be more common in Asia (12, 13). Moreover, there is limited data about the low-dose of NOACs from mainland of China.

As the prescribing patterns and factors driving treatment choice may be evolving, the present cross-sectional study of prescription data has the following objectives: firstly, to describe the prescription pattern of OACs from January 2016 to March 2021 and the factors driving these changes, and secondly, to assess the prevalence of low-dose NOACs and explore the possible associated factors for AF patients.

## Materials and methods

### Data source

Data was extracted from the Hospital Prescription Cooperation Project database, which aimed to promote the rational use of medicines in hospitals of China (14). The database consisted of prescriptions of randomly selected 10 workdays each month from contracted hospitals. Until 2021, there were a total of 134 hospitals from 9 cities (Beijing, Chengdu, Guangzhou, Hangzhou, Shanghai, Tianjin, Zhengzhou, Shenyang and Harbin) enrolled in this project. The study was approved by the *Clinical Research Ethics Committee of Beijing Friendship Hospital* (Ethics approval number: 2021-P2-218-01). Patients’ names and IDs were deleted during data analysis.

### Data collection

The study included prescriptions of patients diagnosed with AF and prescribed with OACs between January 2016 and March 2021 ignoring the age. In this study, OACs included warfarin and all approved NOACs in China, involving dabigatran, rivaroxaban, apixaban and edoxaban.

We also collected the following information for analysis: identification number of patients, sex, age, city, date of prescription, health insurance (with or without), type of visit (outpatient or inpatients), level of hospitals, diagnoses as well as generic names, dosage and administration of all agents in these prescriptions. As the database was recorded quarterly, patients with different OACs owing to the changeover are counted more than once.

### Outcomes and definition of variables

The primary outcome was the changing percentage of different OACs. The secondary outcomes were frequencies as well as factors with the choice of different OACs and dosage of NOACs.

The type of health insurance was classified into two classes according to whether they have or not, without considering the insurance payer. To analyze the effect of age on the predefined outcomes, we classified the age into five groups: 0–17, 18–59, 60–74, 75–89 as well as ≥90 years old and age above 125 were deemed as mistyping and deleted from the related analysis. Prescriptions with missing data on age, health insurance or gender were excluded from related subgroup analysis, but counted in the overall analyze when these factors were not needed. In this study, we defined the dosage of NOACs in Supplementary Table 1 according to the updated guidelines (9). The low dose referred to the low daily dose, which meant that even though rivaroxaban was recommended as 20 mg QD, the “10 mg BID” was also deemed as standard dose. The dosage of warfarin was not analyzed in this study because the intensity of warfarin was determined by INR, which was not available in this database. Concomitantly used drugs assessed in this study includes antiplatelet agents, non-steroidal anti-inflammatory drugs (NSAIDs), antacids as well as steroids.

### Statistical analysis

All continuous variables were presented as mean with standard deviation and compared by *t*-test when normally distributed, or reported as medians with interquartile ranges (IQRs) and compared with the Wilcoxon test when skewed. Categorical variables were shown as frequencies and percentages, and evaluated by either chi-square tests. Multivariate logistic regressions were conducted to examine factors associated with the choice of OACs and dosage of NOACs, results were shown with odds ratios (OR) and 95% confidence intervals (CIs). All statistical analyses were performed using SAS software version 9.4 (SAS Institute, Cary, North Carolina). The two-sided *P* < 0.05 was considered statistically significant.

## Results

### Study population

**FIGURE 1:**
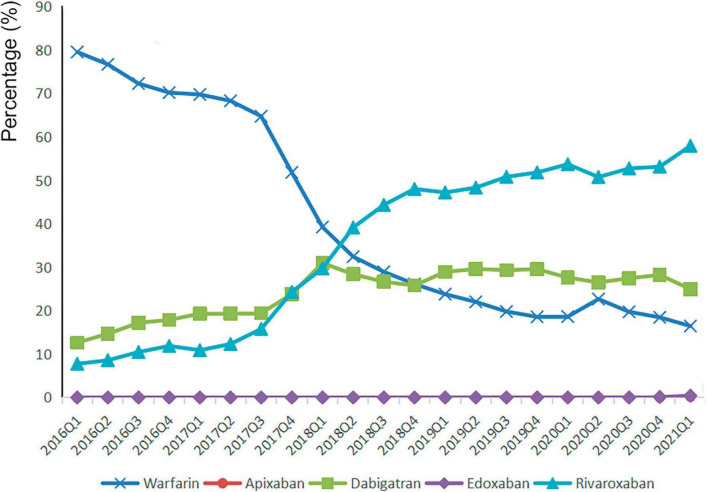
The percentage changes of oral anticoagulants over time.

Between January 2016 and March 2021, there were 220,083 distinct prescriptions diagnosed with AF patients and prescribed with OACs. Figure 1 displayed the changing trends of OACs: the portion of NOACs increased, while that of warfarin decreased over years. The percentage of NOACs exceeded the warfarin from 2018 Q1, and warfarin accounted for only 16.47% of all included AF patients in 2021 Q1. Table 1 showed the baseline characteristics for all included patients by treatment group. Patients prescribed with NOACs and warfarin were significantly different in age, gender, source of data, health insurance, levels of hospitals, comorbidities, concomitant use of drugs as well as the geographical location of patients. The percentage changes of NOACs over time under different conditions can be seen in Supplementary Figure 1.

**TABLE 1:** Baseline characteristics of included atrial fibrillation patients with oral anticoagulants.

	NOAC (*N* = 154,787)	Warfarin (*N* = 65,296)	Overall (*N* = 220,083)	*P*
Age (medium, IQR), years	69 (61, 76)	73 (64, 81)	71 (63, 80)	<0.001*
Age groups (*N*, %), years				<0.001*
0–17	12 (0.01)	12 (0.02)	24 (0.01)	
18–60	23,126 (15.2)	13,363 (20.98)	36,489 (16.91)	
60–74	61,842 (40.66)	30,992 (48.66)	92,834 (43.02)	
75–89	61,542 (40.46)	18,674 (29.32)	80,216 (37.17)	
> = 90	5,577 (3.67)	646 (1.01)	6,223 (2.88)	
Male (*N*, %)	82,666 (54.70)	32,908 (51.70)	115,574 (53.81)	<0.001*
Outpatients	119,774 (77.38)	39,880 (61.08)	159,654 (72.54)	<0.001*
Medicare holder (*N*, %)	121,122 (87.63)	50,229 (85.05)	171,351 (86.86)	<0.001*
Geographical location (*N*, %)				<0.001*
Beijing	43,580 (28.15)	13,873 (21.25)	57,453 (26.11)	
Chengdu	9,404 (6.08)	10,549 (16.16)	19,953 (9.07)	
Guangzhou	24,228 (15.65)	5,952 (9.12)	30,180 (13.71)	
Harbin	3185 (2.06)	1,756 (2.69)	4,941 (2.25)	
Hangzhou	14,560 (9.41)	11,266 (17.25)	25,826 (11.73)	
Shanghai	42,555 (27.49)	16,517 (25.3)	59,072 (26.84)	
Shenyang	8,853 (5.72)	2,141 (3.28)	10,994 (5)	
Tianjin	6,605 (4.27)	1,498 (2.29)	8,103 (3.68)	
Zhengzhou	1,817 (1.17)	1,744 (2.67)	3,561 (1.62)	
Levels of hospitals (*N*, %)				<0.001*
1	489 (0.32)	69 (0.11)	558 (0.25)	
2	6,954 (4.49)	3,743 (5.73)	10,697 (4.86)	
3	147,344 (95.19)	61,484 (94.16)	208,828 (94.89)	
Comorbidities (*N*, %)				
Hypertension	46,241 (29.87)	17,500 (26.8)	63,741 (28.96)	<0.001*
Diabetes	11,212 (7.24)	4,137 (6.34)	15,349 (6.97)	<0.001*
Heart failure	14,922 (9.64)	12,746 (19.52)	27,668 (12.57)	<0.001*
ACS	35,792 (23.12)	12,644 (19.36)	48,436 (22.01)	<0.001*
Stroke	12,174 (7.87)	5,269 (8.07)	17,443 (7.93)	0.10
DVT	612 (0.4)	56 (0.09)	668 (0.3)	<0.001*
PE	765 (0.49)	274 (0.42)	1,039 (0.47)	0.02*
PAD	870 (0.56)	270 (0.41)	1140 (0.52)	<0.001*
Liver disease	645 (0.42)	381 (0.58)	1026 (0.47)	<0.001*
Kidney disease	1,370 (0.89)	840 (1.29)	2,210 (1)	<0.001*
Peptic ulcer	747 (0.48)	190 (0.29)	937 (0.43)	< 0.001*
Concomitant drugs (*N*, %)				
Antiplatelet agents	6,923 (4.47)	3,500 (5.36)	10,423 (4.74)	<0.001*
Antacids	27,277 (17.62)	13,153 (20.14)	40,430 (18.37)	<0.001*
NSAIDs	192 (0.12)	236 (0.36)	428 (0.19)	<0.001*
Steroids	2,007 (1.30)	3,090 (4.73)	5,097 (2.32)	<0.001*

*Results were significantly different between groups. ACS, acute coronary syndrome; DVT, deep venous thrombosis; PE, pulmonary embolism; PAD, peripheral arterial disease; NSAIDs, non-steroidal anti-inflammatory drugs; NOACs, non-vitamin K antagonist oral anticoagulants.

**FIGURE 2:**
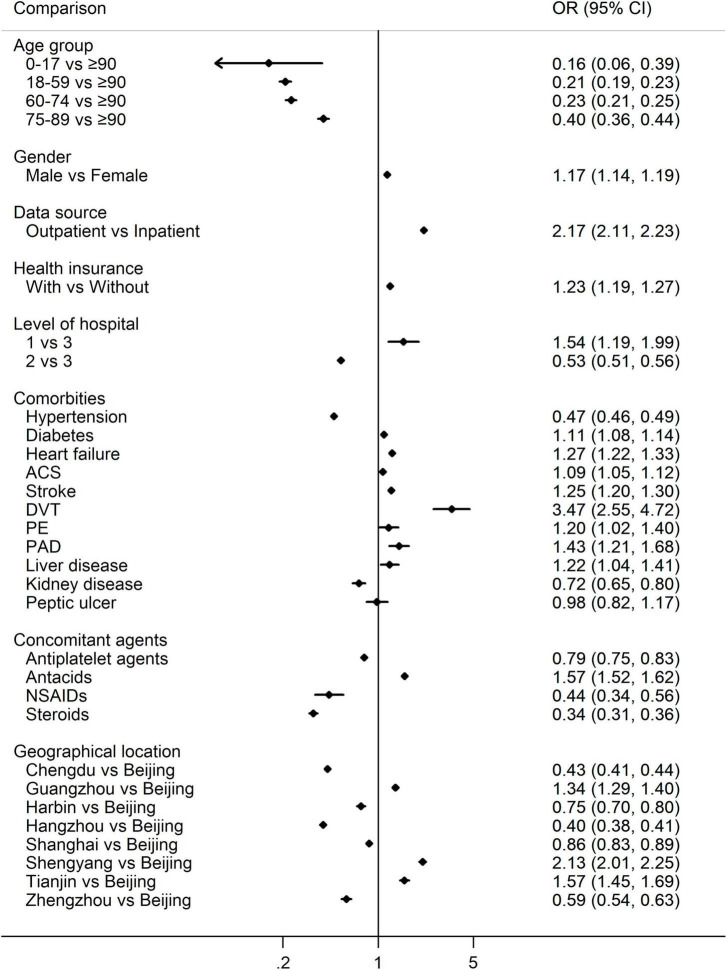
Multivariate logistic regression for the percentage of NOACs vs. warfarin. OR > 1 indicates more NOACs, while OR < 1 indicates more warfarin. NOACs, non-vitamin K antagonist oral anticoagulants; OR, odds ratio; ACS, acute coronary syndrome; DVT, deep venous thrombosis; PE, pulmonary embolism; PAD, peripheral arterial disease; NSAIDs, non-steroidal anti-inflammatory drugs.

Multi-variate logistic regression suggested that the usage of NOAC was more frequent than warfarin for the male (vs. female, OR 1.17, 95% CI 1.14–1.19), the elderly (*P* < 0.001 among age groups), outpatients (vs. inpatients, OR 2.17, 95% CI 2.11–2.23), health insurance holder (vs. without, OR 1.23, 95% CI 1.19–1.27), patients treated in the primary hospitals (vs. tertiary hospitals OR 1.54, 95% CI 1.19–1.99), and diagnosed with ACS (OR 1.09, 95% CI 1.05–1.12), stroke (OR 1.25, 95% CI 1.20–1.30), heart failure (OR 1.27, 95% CI 1.22–1.33), deep venous thrombosis (DVT, OR 3.47, 95% CI 2.55–4.72), diabetes (OR 1.11, 95% CI 1.08–1.14), peripheral arterial disease (PAD, OR 1.43, 95% CI 1.21–1.68), pulmonary embolism (PE, OR 1.20, 95% CI 1.02–1.40), liver disease (OR 1.22, 95% CI 1.04–1.41), and co-administration of antacids (OR 1.57, 95% CI 1.52–1.62). On the contrary, NOACs were less common in patients diagnosed with hypertension (OR 0.47, 95% CI 0.46–0.49), kidney disease (OR 0.72, 95% CI 0.65–0.80), and concomitant administration of antiplatelet agents (OR 0.79, 95% CI 0.75–0.83), NSAIDs (OR 0.44, 95% CI 0.34–0.56) as well as steroids (OR 0.34, 95% CI 0.31–0.36) (Figure 2).

For patients prescribed with NOACs, the frequencies of apixaban, rivaroxaban, edoxaban and dabigatran were also different in age, gender, source of data, health insurance holder, levels of hospitals, comorbidities, concomitant use of drugs as well as the geographical location of patients, details can be seen in Supplementary Table 2. Rivaroxaban (62.25%) and dabigatran (37.65%) were the most commonly used, accounting for 99.9% of all NOAC prescriptions.

**FIGURE 3:**
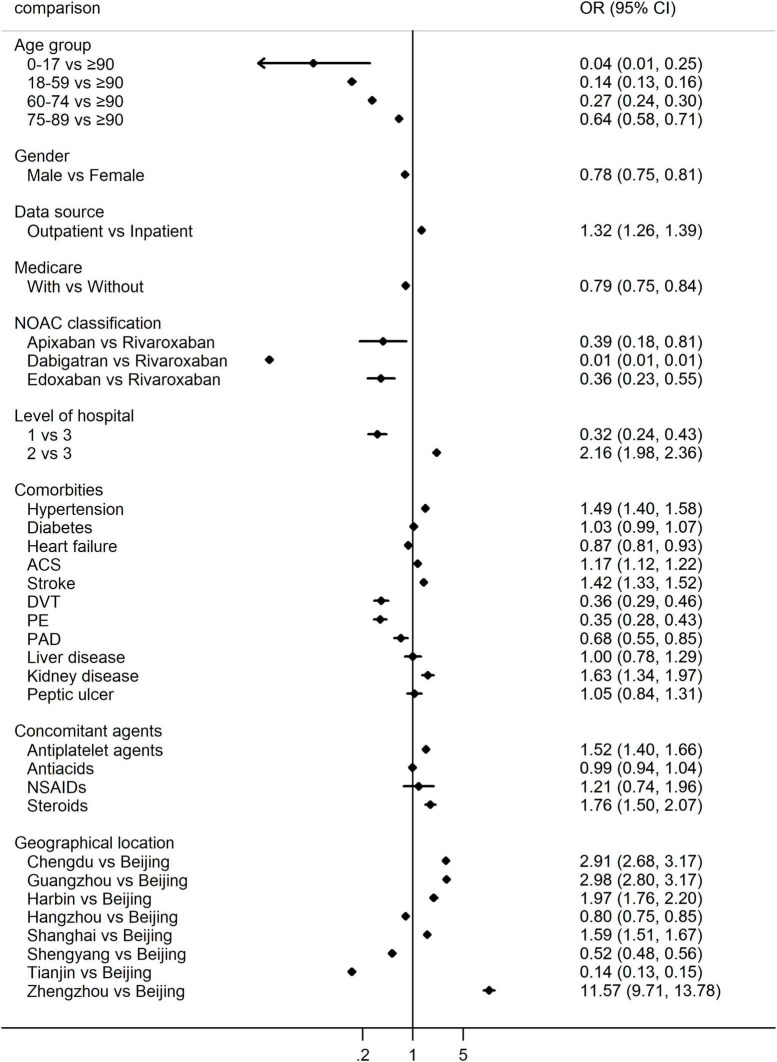
Multivariate logistic regression for the percentage of low-dose vs. standard dose of NOACs. OR > 1 indicates more low-dose of NOACs, while OR < 1 indicates more standard dose of NOACs. NOACs, non-vitamin K antagonist oral anticoagulants; OR, odds ratio; ACS, acute coronary syndrome; DVT, deep venous thrombosis; PE, pulmonary embolism; PAD, peripheral arterial disease; NSAIDs, non-steroidal anti-inflammatory drugs.

When considering the intensity of NOACs as predefined, we found that 44.54% of patients were prescribed low-dose of NOACs, and the changing trends over years were demonstrated in Supplementary Figure 2. The choice of low vs. standard dose of NOACs was affected by age, gender, source of data, health insurance, levels of hospitals, comorbidities, concomitant use of drugs as well as the geographical location of patients, details can be seen in Supplementary Table 3. Multivariate logistic regression taking these factors into account found that the frequency of low-dose of NOACs increased with age, and was significantly different among different geographic locations, and levels of hospitals (*P* < 0.001 within groups, Figure 3). Low-dose of NOACs were more in the outpatients (OR 1.32, 95% CI 1.26–1.39), suffering from hypertension (OR 1.49, 95% CI 1.40–1.58), ACS (OR 1.17, 95% CI 1.12–1.22), stroke (OR 1.42, 95% CI 1.33–1.52) and kidney disease (OR 1.63, 95% CI 1.34–1.97), and concomitantly using antiplatelet agents (OR 1.52, 95% CI 1.40–1.66) and steroids (OR 1.76, 95% CI 1.50–2.07). On the contrary, low-dose of NOACs were less common in the male (OR 0.78, 95% CI 0.75–0.81), health insurance holder (OR 0.79, 95% CI 0.75–0.84), patients taking apixaban (vs. rivaroxaban, OR 0.39, 95% CI 0.18–0.81), dabigatran (vs. rivaroxaban, OR 0.01, 95% CI 0.01–0.01), edoxaban (vs. rivaroxaban, OR 0.36, 95% CI 0.23–0.55), diagnosed with heart failure (OR 0.87, 95% CI 0.81–0.93), DVT (OR 0.36, 95% CI 0.29–0.46), PE (OR 0.35, 95% CI 0.28–0.43), and PAD (OR 0.68, 95% CI 0.55–0.85) (Figure 3).

**FIGURE 4:**
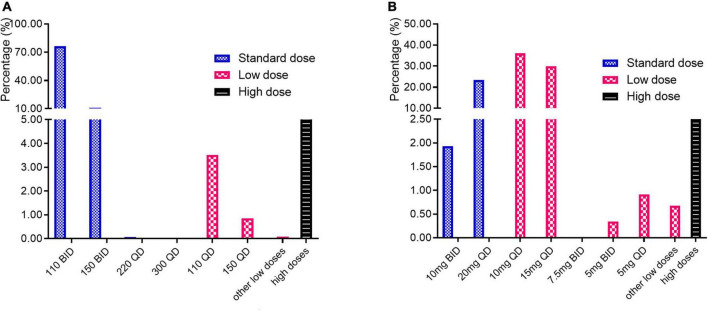
Dosage distribution of **(A)** dabigatran; **(B)** rivaroxaban.

The majority of patients (87.48%) were taking standard dose of dabigatran, with 110 mg BID and 150 mg BID taking account for 76.31 and 11.10%, respectively. Among these patients, some (0.07%) took the standard daily dose of 220 or 300 mg once a day. The low dosages included 110 mg QD (3.51%), 150 mg QD (0.85%) and other low-dose strategies such as 55 mg BID, 75 mg BID, etc. (Figure 4A).

As Figure 4B showed, the low dosage was much more frequent for patients taking rivaroxaban (67.98%). The low daily dose varied from 1 to 15 mg, in which 10 mg QD and 15 mg QD were mostly used, taking account for 36.07 and 29.96% of all rivaroxaban patients, respectively. A total of 25.29% of patients took the standard daily dose of rivaroxaban. Among them, a small proportion (1.93%) took the dosage twice a day.

## Discussion

In this analysis of national electronic prescriptions between 2016 and 2021, we found that among patients with OACs, the percentage of NOACs increased over years, and reached 83.53% of all OAC patients. The prescription of NOACs vs. warfarin was affected by age, gender, health insurance, geographical distributions, comorbidities as well as co-administrated drugs. The low dosage was common for NOACs, especially for rivaroxaban, of which the low dosage accounted for 67.98%.

Though warfarin has been used for many years for the prevention of stroke for NVAF patients (15), its effect is easily affected by food and drugs (16). The time within the therapeutic range (TTR), the time spent achieving an international normalized ratio (INR) of 2-3.0, is used to estimate the quality of anticoagulation during warfarin therapy (4, 5). Some data suggested that if the TTR was less than 60%, the benefit of warfarin may lose (17). The reported TTR during general practice was 25–65%, and varied among patients with different physician specialties, geographical regions and population groups (5, 18). The estimated mean TTR from a tertiary hospital was 49.8% in China (19). As the evidence of NOACs accumulated, NOACs were recommended for NVAF patients and became the first-line strategy over warfarin since 2018 in China (9). From the increasing trend of NOACs and the reversal of percentage vs. warfarin in 2018, we can speculate that Chinese physicians can follow the guideline timely and closely.

It is reported that, compared with men, women have older age, more comorbidities and worse outcomes of stroke (20). Even worse, the female were reported to spend more time to arrive hospitals and receive acute stroke treatments (21–23) though possessing better knowledge of major stroke symptoms and stroke risk factors (24). In this study, we found that the female was less likely to be prescribed with NOACs than warfarin. The higher risk of stroke and lower percentage of NOAC usage calls for more attempts to improve the management of women with NVAF in the future.

In a nationwide cohort study of AF patients ≥75 years in Norway, researchers found that compared with warfarin, both standard and reduced dose of NOACs were associated with similar risks of stroke/systemic embolism as well as lower or similar risks of bleeding (25). The prescription of NOACs increased with age, indicating that physicians in China were accepting NOACs as safe options for elderly patients. Consistent with the worldwide observational cohort study (26), the percentage of NOACs was highest in community hospitals, namely primary hospitals in this study, and demonstrated significant geographic variability.

In this cross-sectional study, we found patients with antiplatelet agents were more prescribed warfarin. For NVAF patients undergoing percutaneous coronary intervention, though NOACs were recommended over VKA as the OAC agent in the US (27), the updated Chinese guideline didn’t give specific recommendations on OAC class owing to the lack of evidence for Chinese (28). Interestingly, we found that NSAIDs and steroids, which can cause gastrointestinal damage, were more co-administrated with warfarin. On the contrary, antacids, which were used to ameliorate gastrointestinal symptoms or injuries, were more co-administrated with NOACs. Further studies were needed to investigate the possible mechanism.

In this study, we found that patients with kidney disease were prescribed less percentage of NOACs vs. warfarin. This might be explained by the fact that the usage of NOACs is contraindicated for patients suffering from severe renal dysfunction during general practice and they were also excluded from trials (3, 4, 29). Though the high incidence of renal impairment during heart failure exacerbation or treatment (30, 31), single-/high-dose of NOACs were reported to reduce the risk of stroke or systemic embolic events by 14% (32) for patients with heart failure. In our study, AF patients suffering from heart failure were more commonly prescribed with NOACs.

In this study, rivaroxaban and dabigatran were the most commonly selected NOACs. Though the ARISTOTLE trial demonstrated its superiority to warfarin in hemorrhagic stroke as well as intracranial and major bleeding (5), it was not approved for stroke prevention for AF patients in China until now. The low percentage of edoxaban might be because it was approved in December 2018, much later than the other two NOACs. As there are no trials to compare different NOACs head-to-head, physicians need to choose one NOAC agent under comprehensive consideration of patients’ conditions such as personal will, age, bleeding risk and dyspepsia symptoms (33).

Though growing evidence showed that low-dose of NOACs was associated with increased risk for adverse events (10, 34, 35), it is commonly used in daily practice, especially for the Asian (12, 36). Similarly, nearly half of patients in this study are receiving low-dose of NOACs. This prescribing pattern might be caused by the lack of effective testing methods to verify the effectiveness of NOACs (37) and the fear of bleeding. Though NOACs have predictable pharmacokinetic and pharmacodynamic profiles, physicians may prefer to adjust doses to protect patients against bleeding risk in some “complicated” situations. Furthermore, we have to note that the Xa antidote was not available in China, once major bleeding occurs, patients have to use prothrombin complex concentrates or recombinant factor VIIa (38, 39), which is very expensive. Idarucizumab, the monoclonal antibody to reverse the anticoagulant effect of dabigatran (40), is also expensive in China.

Consistent with the previous study (36), we found that the low dosage was extremely common for rivaroxaban. This might be caused that rivaroxaban is a regular formulation and can be divided as needed. On the contrary, the oral bioavailability of dabigatran etexilate increases by 75% when the pellets are taken without the capsule shell compared to the intact capsule formulation. And the label of dabigatran declares that PRADAXA capsules should not be broken, chewed, or opened before administration (41). However, we can see that there were still a small proportion of patients prescribed with 55 mg BID or 75 mg BID with the 110 mg and 150 mg strengths, respectively. More efforts should be taken to improve the physician’s knowledge about medication.

In this study, we found that the predictors of low-dose of NOACs were female sex, outpatients, without health insurance, concomitantly use of antiplatelet agents and steroids, diagnosed with hypertension, ACS, stroke and kidney disease. This is not the same with results from GARFIELD-AF, the global prospective cohort study, in which diabetes was also detected to be a predictor of low-dose of NOACs (42).

To our knowledge, this is the first study to describe the prescribing pattern of OACs and their associated factors for AF patients until 2021 in mainland of China. Of course, we acknowledge the following limitations: First of all, the CHA2DS2-VASc score was not calculated in this study. However, we have assessed the affection of all involved factors in the CHA2DS2-VASc score on the selection of OACs and dosage of NOACs. On the other hand, the CHA2DS2-VASc score based on outpatient prescriptions might be underestimated because of the missing data. Secondly, we cannot predict the rate of AF patients taking OACs because patients may not need to prescribe OACs for each visit. Similarly, owing to the lack of continuous drug use by patients in this database, we cannot assess the interchange among OACs. Thirdly, because of the nature of this database, we didn’t use the ICD10 code to extract diagnosis information. However, we have got all the possible expressions for each diagnose from the database organizer to reduce omissions. At the same time, we didn’t specify the NVAF and valve AF because we are afraid of data omission.

## Conclusion

In conclusion, the percentage of NOACs increase over years for patients taking OACs between 2016 and 2021. The prescription of NOACs vs. warfarin was overall complying with updated evidence and affected by age, gender, health insurance, geographic distributions, comorbidities as well as concomitant drugs. Low-dose of NOACs was common in general practice, especially for rivaroxaban. Further studies were needed to assess the possible factors.

## Data availability statement

The original contributions presented in the study are included in the article/Supplementary material, further inquiries can be directed to the corresponding authors.

## Ethics statement

The studies involving humans were approved by the Clinical Research Ethics Committee of Beijing Friendship Hospital (Ethics approval number: 2021-P2-218-01). The studies were conducted in accordance with the local legislation and institutional requirements. Written informed consent for participation was not required from the participants or the participants’ legal guardians/next of kin in accordance with the national legislation and institutional requirements.

## Author contributions

ML: Data curation, Software, Writing – original draft. SZ: Investigation, Methodology, Writing – original draft. LW: Methodology, Software, Writing – review and editing. LH: Investigation, Writing – original draft. DL: Data curation, Validation, Writing – review and editing. JL: Methodology, Project administration, Writing – review and editing. YL: Supervision, Writing – review and editing.
