# Supplementary material for: Real-world national trends and influencing factors preference of non-vitamin K antagonist oral anticoagulants in China
Source: Front Med (Lausanne). 2023 Nov 21;10:1258536. doi: 10.3389/fmed.2023.1258536 (PMC10702983; doi:10.3389/fmed.2023.1258536)

**Real-world national trends and influencing factors preference of Non-vitamin K antagonist oral anticoagulants in China**

Supplemental data

Table S1. Definitions on dosages of NOACs in this study

Table S2. Characteristics of patients with NOACs

Table S3. Characteristics of patients with different dosages of NOACs.

Figure S1. The percentage changes of NOACs over time under different conditions.

Figure S2. Percentage changes about the dosage of NOACs over time.

Table S1. Definitions on dosages of NOACs in this study

|  | Standard dosage | Low dosage | High dosage |
| --- | --- | --- | --- |
| Dabigatran | 300mg or 220mg per day | <220mg per day | >300mg per day |
| Rivaroxaban | 20mg per day | <20mg per day | >20mg per day |
| Apixaban | 5mg bid | 2.5mg bid | >10mg per day |
| Edoxaban | 60mg qd | 30mg qd | >60mg per day |

NOACs: non-vitamin K antagonist oral anticoagulants

Table S2. Characteristics of patients with NOACs

|  | Overall (N=154,787) | Apixaban (N=53) | Dabigatran (N=58,277) | Edoxaban (N=96) | Rivaroxaban (N=96,361) | P |
| --- | --- | --- | --- | --- | --- | --- |
| Age groups (N,%),years |  |  |  |  |  | <0.001^*^ |
| 0-17 | 12 (0.01) | 0 (0) | 6 (0.01) | 0 (0) | 6 (0.01) |  |
| 18-60 | 23,126 (15.2) | 8 (17.39) | 9,576 (16.76) | 25 (26.04) | 13,517 (14.26) |  |
| 60-74 | 61,842 (40.66) | 21 (45.65) | 23,793 (41.63) | 40 (41.67) | 37,988 (40.07) |  |
| 75-89 | 61,542 (40.46) | 16 (34.78) | 22,146 (38.75) | 29 (30.21) | 39,351 (41.51) |  |
| >=90 | 5,577 (3.67) | 1 (2.17) | 1,627 (2.85) | 2 (2.08) | 3,947 (4.16) |  |
| Male (N, %) | 82,666 (54.7) | 30 (57.69) | 31,819 (55.31) | 60 (62.5) | 50,757 (54.31) | <0.001^*^ |
| Outpatients | 119,774 (77.38) | 33 (62.26) | 43,756 (75.08) | 86 (89.58) | 75,899 (78.77) | <0.001^*^ |
| Medicare holder (N,%) | 121,122 (0.88) | 41 (0.82) | 42,763 (0.84) | 46 (0.48) | 78,272 (0.9) | <0.001^*^ |
| Geographical location (N, %) |  |  |  |  |  |  |
| Beijing | 43,580 (28.15) | 1 (1.89) | 19,255 (33.04) | 94 (97.92) | 24,230 (25.15) | <0.001^*^ |
| Chengdu | 9,404 (6.08) | 0 (0) | 3,529 (6.06) | 0 (0) | 5,875 (6.1) |  |
| Guangzhou | 24,228 (15.65) | 14 (26.42) | 9,102 (15.62) | 0 (0) | 15,112 (15.68) |  |
| Harbin | 3,185 (2.06) | 1 (1.89) | 1,275 (2.19) | 0 (0) | 1,909 (1.98) |  |
| Hangzhou | 14,560 (9.41) | 23 (43.4) | 5,398 (9.26) | 0 (0) | 9,139 (9.48) |  |
| Shanghai | 42,555 (27.49) | 2 (3.77) | 13,586 (23.31) | 0 (0) | 28,967 (30.06) |  |
| Shenyang | 8,853 (5.72) | 5 (9.43) | 4,520 (7.76) | 0 (0) | 4,328 (4.49) |  |
| Tianjin | 6,605 (4.27) | 1 (1.89) | 557 (0.96) | 0 (0) | 6,047 (6.28) |  |
| Zhengzhou | 1,817 (1.17) | 6 (11.32) | 1,055 (1.81) | 2 (2.08) | 754 (0.78) |  |
| Levels of hospitals (N, %) |  |  |  |  |  | <0.001^*^ |
| 1 | 489 (0.32) | 0 (0) | 282 (0.48) | 0 (0) | 207 (0.21) |  |
| 2 | 6,954 (4.49) | 0 (0) | 2,939 (5.04) | 0 (0) | 4,015 (4.17) |  |
| 3 | 147,344 (95.19) | 53 (100) | 55,056 (94.47) | 96 (100) | 92,139 (95.62) |  |
| Comorbidities (N, %) |  |  |  |  |  |  |
| Hypertension | 46,241 (29.87) | 17 (32.08) | 17,405 (29.87) | 65 (67.71) | 28,754 (29.84) | <0.001^*^ |
| Diabetes | 11,212 (7.24) | 3 (5.66) | 4,550 (7.81) | 20 (20.83) | 6,639 (6.89) | <0.001^*^ |
| Heart failure | 14,922 (9.64) | 3 (5.66) | 5,180 (8.89) | 21 (21.88) | 9,718 (10.08) | <0.001^*^ |
| ACS | 35,792 (23.12) | 5 (9.43) | 13,416 (23.02) | 38 (39.58) | 22,333 (23.18) | <0.001^*^ |
| Stroke | 12,174 (7.87) | 5 (9.43) | 5,051 (8.67) | 3 (3.13) | 7,115 (7.38) | <0.001^*^ |
| DVT | 612 (0.4) | 0 (0) | 102 (0.18) | 1 (1.04) | 509 (0.53) | <0.001^*^ |
| PE | 765 (0.49) | 0 (0) | 196 (0.34) | 0 (0) | 569 (0.59) | <0.001^*^ |
| PAD | 870 (0.56) | 0 (0) | 217 (0.37) | 0 (0) | 653 (0.68) | <0.001^*^ |
| Liver disease | 645 (0.42) | 0 (0) | 244 (0.42) | 1 (1.04) | 400 (0.42) | 0.77^*^ |
| Kidney disease | 1,370 (0.89) | 0 (0) | 420 (0.72) | 0 (0) | 950 (0.99) | <0.001^*^ |
| Peptic ulcer | 747 (0.48) | 0 (0) | 339 (0.58) | 0 (0) | 408 (0.42) | <0.001^*^ |
| Concomitant drugs (N, %) |  |  |  |  |  |  |
| Antiplatelet agents | 6,923 (4.47) | 1 (1.89) | 2,139 (3.67) | 3 (3.13) | 4,780 (4.96) | <0.001^*^ |
| Antacids | 27,277 (17.62) | 20 (37.74) | 11,271 (19.34) | 7 (7.29) | 15,979 (16.58) | <0.001^*^ |
| NSAIDs | 192 (0.12) | 0 (0) | 52 (0.09) | 0 (0) | 140 (0.15) | 0.02^*^ |
| Steroids | 2,007 (1.3) | 3 (5.66) | 647 (1.11) | 2 (2.08) | 1,355 (1.41) | <0.001 |

* Results were significantly different between groups. ACS: acute coronary syndrome; DVT: deep venous thrombosis; PE: pulmonary embolism; PAD: peripheral arterial disease; NSAIDs: nonsteroidal anti-inflammatory drugs; NOACs: non-vitamin K antagonist oral anticoagulants

Table S3. Characteristics of patients with different dosages of NOACs

|  | Low dose (N=68,943) | Standard dose (N=76,533) | Overall (N=145,476) | P |
| --- | --- | --- | --- | --- |
| Age groups (N,%),years |  |  |  | <0.001^*^ |
| 0-17 | 2 (0) | 9 (0.01) | 11 (0.01) |  |
| 18-60 | 6,749 (9.9) | 14,873 (19.56) | 21,622 (14.99) |  |
| 60-74 | 24,876 (36.48) | 33,611 (44.19) | 58,487 (40.54) |  |
| 75-89 | 33,074 (48.5) | 25,777 (33.89) | 58,851 (40.8) |  |
| >=90 | 3,498 (5.13) | 1,786 (2.35) | 5,284 (3.66) |  |
| Male (N, %) | 34,691 (51.45) | 43,601 (57.44) | 78,292 (54.62) | <0.001^*^ |
| Outpatients | 55,785 (80.64) | 59,110 (76.91) | 114,895 (78.68) | <0.001^*^ |
| Medicare holder (N,%) | 57,017 (89.25) | 57,817 (85.82) | 114,834 (87.49) | <0.001^*^ |
| Geographical location (N, %) |  |  |  | <0.001^*^ |
| Beijing | 17,108 (24.81) | 25,417 (33.21) | 42,525 (29.23) |  |
| Chengdu | 5,435 (7.88) | 3,978 (5.2) | 9,413 (6.47) |  |
| Guangzhou | 11,932 (17.31) | 7,975 (10.42) | 19,907 (13.68) |  |
| Harbin | 1,440 (2.09) | 1,705 (2.23) | 3,145 (2.16) |  |
| Hangzhou | 6,412 (9.3) | 7,917 (10.34) | 14,329 (9.85) |  |
| Shanghai | 22,325 (32.38) | 17,443 (22.79) | 39,768 (27.34) |  |
| Shenyang | 2,181 (3.16) | 6,228 (8.14) | 8,409 (5.78) |  |
| Tianjin | 1,439 (2.09) | 5,089 (6.65) | 6,528 (4.49) |  |
| Zhengzhou | 671 (0.97) | 781 (1.02) | 1,452 (1) |  |
| Levels of hospitals (N, %) |  |  |  | <0.001^*^ |
| 1 | 95 (0.14) | 388 (0.51) | 483 (0.33) |  |
| 2 | 3,682 (5.34) | 2,410 (3.15) | 6,092 (4.19) |  |
| 3 | 65,166 (94.52) | 73,735 (96.34) | 138,901 (95.48) |  |
| Comorbidities (N, %) |  |  |  |  |
| Hypertension | 21,326 (30.93) | 23,571 (30.8) | 44,897 (30.86) | 0.56^*^ |
| Diabetes | 4,865 (7.06) | 5,850 (7.64) | 10,715 (7.37) | <0.001^*^ |
| Heart failure | 7,946 (11.53) | 6,352 (8.3) | 14,298 (9.83) | <0.001^*^ |
| ACS | 17,113 (24.82) | 16,996 (22.21) | 34,109 (23.45) | <0.001^*^ |
| Stroke | 5,757 (8.35) | 5,868 (7.67) | 11,625 (7.99) | <0.001^*^ |
| DVT | 264 (0.38) | 313 (0.41) | 577 (0.4) | 0.43 |
| PE | 312 (0.45) | 406 (0.53) | 718 (0.49) | 0.03^*^ |
| PAD | 480 (0.7) | 366 (0.48) | 846 (0.58) | <0.001^*^ |
| Liver disease | 289 (0.42) | 326 (0.43) | 615 (0.42) | 0.85 |
| Kidney disease | 846 (1.23) | 497 (0.65) | 1,343 (0.92) |  |
| Peptic ulcer | 283 (0.41) | 442 (0.58) | 725 (0.5) | <0.001^*^ |
| Concomitant drugs (N, %) |  |  |  |  |
| Antiplatelet agents | 3,694 (5.36) | 2,751 (3.59) | 6,445 (4.43) | <0.001^*^ |
| Antacids | 10,662 (15.46) | 14,140 (18.48) | 24,802 (17.05) | <0.001^*^ |
| NSAIDs | 104 (0.15) | 81 (0.11) | 185 (0.13) | 0.02^*^ |
| Steroids | 1,123 (1.63) | 719 (0.94) | 1,842 (1.27) | <0.001^*^ |
| NOAC class |  |  |  | <0.001^*^ |
| Apixaban | 29 (0.04) | 18 (0.02) | 47 (0.03) |  |
| Dabigatran | 2,635 (3.82) | 51,832 (67.73) | 54,467 (37.44) |  |
| Edoxaban | 46 (0.07) | 48 (0.06) | 94 (0.06) |  |
| Rivaroxaban | 66,233 (96.07) | 24,635 (32.19) | 90,868 (62.46) |  |

* Results were significantly different between groups. ACS: acute coronary syndrome; DVT: deep venous thrombosis; PE: pulmonary embolism; PAD: peripheral arterial disease; NSAIDs: nonsteroidal anti-inflammatory drugs; NOACs: non-vitamin K antagonist oral anticoagulants

Figure S1. The percentage changes of NOACs over time under different conditions


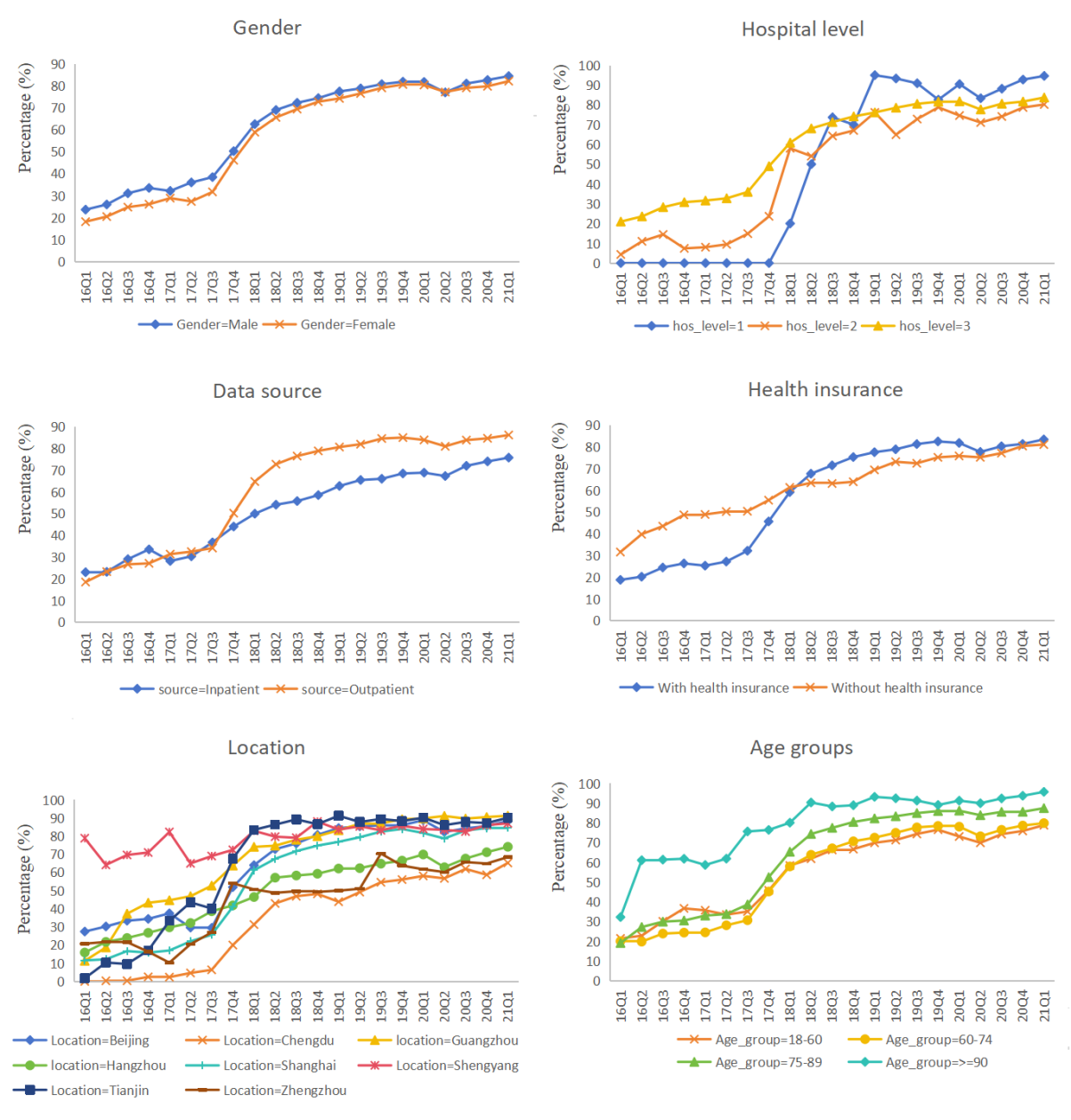


Figure S2. Percentage changes about the dosage of NOACs over time


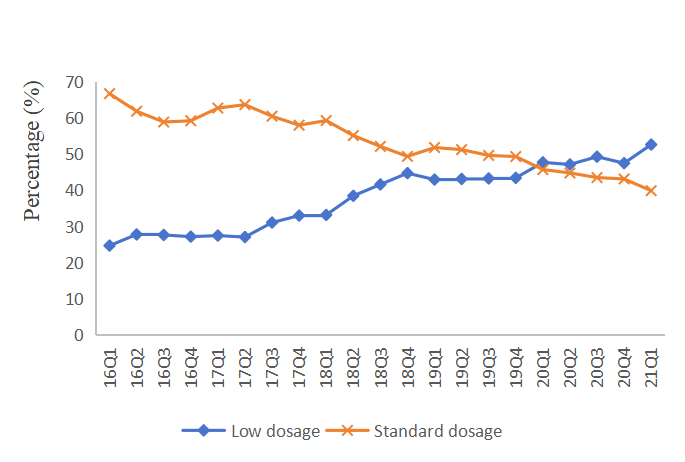

Supplement: Supplementary file 1 [file Table_1.docx]
